# A Unified Method for Triple Oxygen Isotope Analysis of Sulfate, Water, and Organics

**DOI:** 10.1021/acs.analchem.5c06406

**Published:** 2026-03-06

**Authors:** Fabian Zahnow, Dingsu Feng, Andreas Pack, David Bajnai, Daniel Herwartz

**Affiliations:** † Institute of Geosciences, 9142 Ruhr University Bochum, Universitätsstr. 150, Bochum 44801, Germany; ‡ Geoscience Center, 9375 University of Göttingen, Goldschmidtstraße 1, Göttingen 37077, Germany

## Abstract

Triple oxygen isotope analyses of sulfate, water, and organic materials provide vital insights into geochemical processes. Here, we present the fully automated TORCH method (Triple Oxygen isotope analysis using Reduction, Conversion, and High-precision laser spectroscopy), which enables the direct normalization of various materials to the VSMOW-SLAP reference scale using a unified method. The method integrates high-temperature thermal conversion elemental analysis (TC/EA), high-voltage glow discharge CO-to-CO_2_ conversion, and tunable infrared laser direct absorption spectrometry for simultaneous δ^18^O and Δ′^17^O determination from CO_2_. We applied the TORCH method to international and in-house standards of Barite (IAEA-SO-5, IAEA-SO-6), benzoic acid (IAEA-601), cellulose (Sigma-Aldrich 0742213, labeled Cellulose-KOSI), and water (VSMOW2, SLAP2, USGS46), demonstrating quantitative oxygen yields and a reproducibility reaching ±14 per meg for Δ′^17^O and ±0.3‰ for δ^18^O (1 SD). We show that consistent triple oxygen isotope values across different sample types and analytical techniques are achieved when quantitative oxygen extraction and accurate scaling are assured. The TORCH method delivers flexibility across varied matrices, paving the way for high-accuracy triple oxygen isotope analysis of geologically and environmentally relevant sample types.

## Graphical abstract

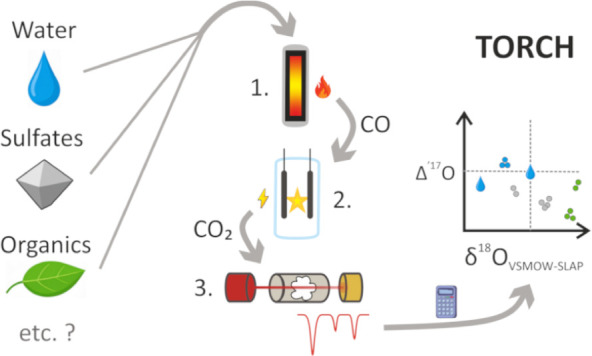

## Introduction

The analysis of all three stable oxygen isotopes (^16^O, ^17^O, ^18^O) is providing a second dimension to the traditional ^18^O/^16^O scale, which is increasingly employed in several research fields.^1^ Established laser fluorination methods enable determination of triple oxygen isotope variations in silicate and oxide samples with reproducibility reaching the lower per meg range.^2–4^ The precise analyses of various materials, however, require material-specific methods. Water samples can be analyzed using several approaches, including Ni bomb combustion,^5,6^ injection into a CoF_3_ reactor,^7,8^ cavity output spectroscopy^9^ or cavity ring-down spectroscopy,^10^ as well as the classic method of CO_2_–H_2_O isotope exchange followed by platinum-catalyzed O_2_–CO_2_ exchange.^11^ In contrast, sulfates prepared using laser fluorination give oxygen yields typically around 25–50%,^12,13^ limiting precision and accuracy as evident from poor interlaboratory comparability of reported triple oxygen isotope standard values.^13–16^

The use of different analytical setups for different sample materials complicates the reporting of data on a common reference scale, even within a single laboratory. Oxygen isotopes are reported relative to the Vienna Standard Mean Ocean Water (VSMOW). A second standard, SLAP (Standard Light Antarctic Precipitation), is commonly used to account for variable scale compression observed for instruments and/or sample preparation systems.^17^ While this concept works well for water samples, which are all analyzed using the same instrumentation and routine, normalizing other materials to the VSMOW-SLAP scale may be flawed if analytical artifacts in one of the setups are not accounted for. We suggest that using the same analytical setup and procedure for water and as many other materials as possible may provide a good approach for accurately intercalating the different materials on the VSMOW-SLAP scale. Here, we test an approach that builds on a new combination of published methods and principally allows analyzing any material for Δ′^17^O that can be converted to CO using a conventional Thermal Conversion Elemental Analysis (TC/EA) setup.

Oxygen isotope ratios are expressed using the δ notation^18^ (eq 1).

1 δ17,18O(permil)=103×((O17,18O16)sample(O17,18O16)standard−1)

We report oxygen isotope values on the VSMOW-SLAP scale by analyzing the reference materials VSMOW2 and SLAP2.^19^ Deviation from a reference line in δ^17^O is expressed in the Δ′^17^O notation (eq 2):^1,15,20,21^

$$\Delta^{\prime 17}O_{0.528}\left(\mathrm{per meg}\right)=\left(10^{3}\times \ln\left(\frac{\delta^{17}O}{10^{3}}+1\right)-0.528\times 10^{3}\times \ln\left(\frac{\delta^{18}O}{10^{3}}+1\right)\right)\times 10^{3}$$ 2

Throughout this study, we report the Δ′^17^O notation in per meg using a reference slope of 0.528 and a zero intercept, typically chosen for water samples^22^ and other terrestrial materials.^15,21^ For SLAP2, we adopt the proposed values of δ^18^O_VSMOW_ = −55.5‰ and δ^17^O_VSMOW_ = −29.709‰, which translate to Δ′^17^O_0.528_ = −11 per meg.^6,15^

Recently, advances have been made in high-temperature reduction techniques that extract sample oxygen, for instance from Barite (BaSO_4_), as CO and/or CO_2_ for triple oxygen isotope analysis.^14,16^ Utilizing high-temperature reduction with TC/EA can also be applied to other chemical sediments, organics, and water.^14^ The extracted CO can be converted to CO_2_ via high-voltage glow discharge,^16,23^ a process more frequently used for δ^18^O^16,23,24^ but not yet common for Δ′^17^O.^16^

Other approaches such as TC/EA-methanation–fluorination (HTC-M-F) transfer the CO to H_2_O, which is subsequently fluorinated to yield O_2_,^14^ while the use of I_2_O_5_ on acidified silica gel (Schutze’s reagent) allows oxidation of CO to CO_2_ on a different pathway.^25,26^ The transfer of CO to CO_2_ can also be achieved via Ni-catalyzed conversion at 350 °C.^27^ Oxygen remains the preferred analyte in triple oxygen isotope analysis of sulfates and organics, whether obtained by direct laser fluorination, methanation, or CO_2_–O_2_ exchange over hot platinum (R-D-E).^12–14,16,25,28,29^

The various techniques report external reproducibility for Δ′^17^O, ranging from ±20 to ±50 per meg for direct laser fluorination,^12,13,15^ ±20 per meg for the I_2_O_5_,^25^ less than ±10 per meg for HTC-M-F,^14^ and ±9 per meg for the R-D-E protocol.^16^ Unless noted otherwise, all reproducibility values are given as 1 standard deviation (1 SD).

A recent development employs CO_2_ as the analyte for the triple oxygen isotope measurement via tunable infrared laser direct absorption spectrometry (TILDAS),^27,30–33^ providing high-precision (better than ±10 per meg) for Δ′^17^O by directly measuring the isotopologues of CO_2_. This avoids additional conversion steps known to induce fractionation.^29^ Importantly, all conversion procedures must achieve quantitative gas yields to avoid isotope fractionation and degraded analytical precision.

Here, we introduce the fully automated and high-precision TORCH method (**T**riple **O**xygen isotope analysis using **R**eduction, **C**onversion, and **H**igh-precision laser spectroscopy). The technique integrates high-temperature TC/EA oxygen extraction, high-voltage glow discharge CO-to-CO_2_ conversion, and TILDAS triple oxygen isotope analysis, minimizing sample preparation and enhancing safety by avoiding the use of fluorinating agents and other hazardous materials. The TORCH approach is benchmarked using international and in-house Barite, water, and organic (i.e., benzoic acid and cellulose) standards, with direct VSMOW2-SLAP2 scaling achieved via the same analytical setup for both water and solids.

## Experimental Section

### Materials

Two international Barite reference standards (IAEA-SO-5 and IAEA-SO-6; International Atomic Energy Agency) and one in-house Barite standard (UGSO-1; 99.99% BaSO_4_, Carl Roth GmbH + Co. KG) were analyzed for their triple oxygen isotope composition. An international benzoic acid standard (IAEA-601) and a cellulose in-house working standard (Cellulose-KOSI; Sigma-Aldrich 0742213, from the Center for Stable Isotope Research and Analysis [KOSI], University of Göttingen) were included to assess method performance for organic matrices. Solid sulfate and organic samples were each mixed with a 2:1 stoichiometric excess of powdered carbon (grain size <125 μm, prepared from crushed TC/EA graphite crucibles), then sealed in 0.02 mL silver capsules (99.99% Ag; IVA Analysentechnik GmbH & Co. KG). Blank tests with empty capsules and capsules containing only powdered carbon were conducted using pressure gauge readings and a downstream quadrupole mass spectrometer (Pfeiffer PrismaPro QME 250) on the helium-fed TC/EA system. Neither the silver capsules nor powdered carbon produced detectable signals for H_2_O, CO, CO_2_, or air. Typical sample quantities corresponded to the oxygen content in 2.5 mg Barite (21.4 μmol O_2_). Sealed capsules were stored at 50 °C for at least one night prior to further preparation.

For waters, international reference samples VSMOW2 and SLAP2 were used for direct scaling and USGS46 water served as a test standard. Approximately 1 μL of water was sealed in 3 mm-long, crimped silver tubes (99.95% Ag, 1.1 mm o.d., 0.75 mm i.d.; Goodfellow GmbH) using a custom semiautomatic filling and crimping station (Figure S1) according to Qi et al.^34^

### Automated Setup for Oxygen Reduction, Conversion, and Isotope Analysis

The fully automated oxygen reduction and conversion line (Figure 1) at the University of Göttingen, Germany, is controlled via a National Instruments LabView interface (Figure S2). Each reduction and conversion sequence required approximately 1.5 h, depending on the initial amount of CO to be converted to CO_2_. Relevant metadata—including pressures, temperatures, and thermal conductivity CO signals—were tracked explicitly for each run and stored in CSV format to facilitate quality control and reproducibility (listed in the Supplementary Data).

**Figure 1:**
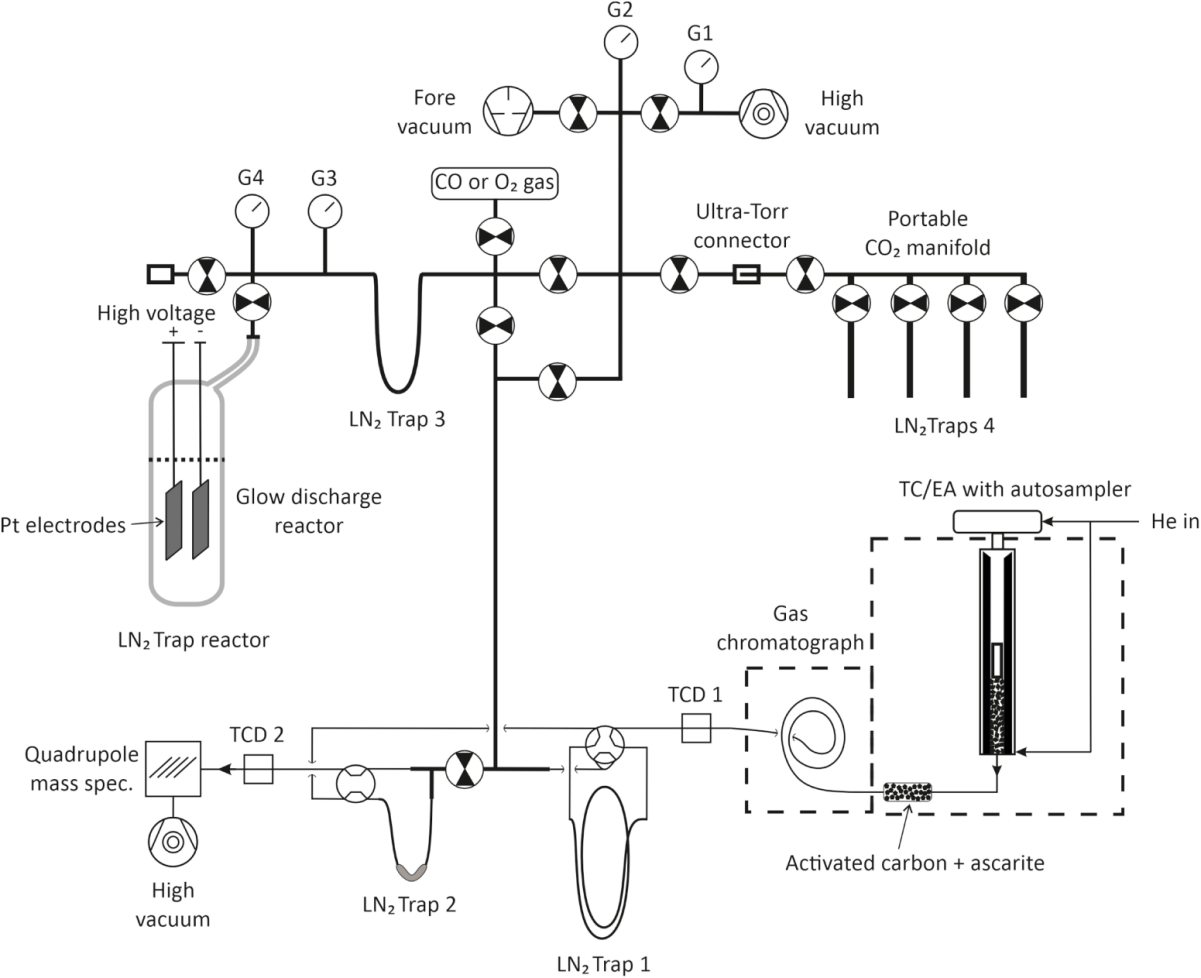
Schematic diagram of the reduction and conversion unit of the TORCH at the University of Göttingen, Germany, for reducing solid or liquid sample material to CO, purification of CO, and subsequent high-voltage glow discharge conversion to CO_2_ for triple oxygen isotope analysis.

### Thermal Conversion for Sample Reduction

Samples, contained in sealed silver capsules or tubes, were introduced into the helium-purged factory-provided autosampler (Thermo Fisher Scientific MAS 200 R) of the TC/EA reduction unit (ThermoFinnigan, Bremen, Germany). No air or contaminant gas was detected from our autosampler. However, future improvements could include adopting a vacuum-pumped zero-blank autosampler. The TC/EA featured a bottom-feed adapter to ensure reverse helium carrier gas flow (60 mL min^–1^) directly through the core of the reactor,^35^ promoting quantitative reduction.

The outer reactor tube was composed of silicon carbide (SiC), which provides a significantly lower CO background compared to the standard Al_2_O_3_ ceramic tube,^36^ especially with increasing reactor temperature (Figure S3). The mass 28 (CO/N_2_) background remained low with the SiC tube, at 1.5 × 10^–12^ A in the quadrupole mass spectrometer and did not increase significantly with temperature. In contrast, the Al_2_O_3_ tube at 1450 °C yielded a mass 28 background an order of magnitude higher (1.4 × 10^–11^ A). The inner tube was made of glassy carbon and filled with glassy carbon granulate up to the reactor’s hot zone, which was maintained at 1450 °C. The granulate was contained by a silver wool plug at the bottom. On top of the hot zone, a graphite crucible prefilled with 5 mg of powdered graphite supported quantitative reduction by providing excess available carbon. The crucible was replaced after a maximum of 16 sample runs.

Once dropped into the hot zone, the sample oxygen was converted to CO and, if present in the sample, hydrogen was released as H_2_ (eq 3). Downstream of the reactor, a trap filled with ascarite and activated carbon removed any acidic byproducts that could damage the column of the gas chromatograph. The gas stream was then passed through the gas chromatograph (1 m, 1/4 in. o.d., 5 mm i.d., filled with 5 Å molecular sieve, operated at 90 °C; heated to 350 °C weekly for cleaning) to separate CO and H_2_.

3 $$H_{2}O+C\overset{1450\;{}^{\circ}C}{\to }\mathrm{CO}+H_{2}$$

While the H_2_ fraction was not analyzed here, the setup allows for future hydrogen isotope (δ^2^H) measurements (Figure S2, Trap 9). The individual vacuum- and helium-fed parts of the reduction line were tested for air leakage prior to the installation of gas-removing components such as the gas chromatography column or the ascarite trap.

The gas stream was further purified by sequential LN_2_-cooled U-traps (Trap 1 for moisture and Trap 2 for collecting the CO fraction using a 5 Å molecular sieve). A thermal conductivity detector (TCD) and the quadrupole mass spectrometer at the helium exhaust continuously monitored the gas composition.

### Glow Discharge for CO_2_ Production

The purified sample CO, collected on Trap 2, was expanded into the glow discharge reactor on the vacuum side of the experiment (Figure S4). Trap 2 was heated to 65 °C to ensure the complete release of CO from the molecular sieve during the glow discharge. The effective CO yield (i.e., the yield of the TC/EA reduction) was calculated from the expected stoichiometric versus actual pressure (G4, Figure 1) in the glow discharge volume. The glow discharge volume was calibrated with defined amounts of CO, inserted into the volume via a syringe through a septum attached to the line. The glow discharge reactor and an additional U-trap (Trap 3, positioned between the reactor and Trap 2) were immersed in LN_2_. Trap 3 ensured that the produced CO_2_ did not back-transfer to the molecular sieve if it was not instantly frozen in the discharge reactor.

Glow discharge, converting CO to CO_2_ (eq 4), was initiated by applying a high voltage (up to 5 kV) between two platinum electrodes (5 × 1 cm), once the system reached LN_2_ temperature. The actual discharge voltage varied from ∼0.4 kV at initial pressures of ∼2.5 mbar to up to 3 kV as pressure decreased to 0.04 mbar, until the discharge extinguished below ∼0.02–0.03 mbar (Figure S5). After conversion, residual CO was pumped away, and CO_2_ was transferred to a 4-port manifold for eventual triple oxygen isotope measurement. Tests to thaw and refreeze CO_2_ to recover trapped CO were found unnecessary, as this did not yield significant additional CO to reignite the glow discharge (∼0.01 mbar ≙ 0.3% yield) nor altered the isotopic composition.

4 $$2\mathrm{CO}\overset{\leq 5\mathrm{kV}}{\to }C+{\mathrm{CO}}_{2}$$

The electrodes and reactor glass were carefully polished after 16 sample runs. Inner walls and anode were cleaned with powdered Al_2_O_3_, while the carbon-coated cathode was treated with fine-grained abrasive paper before polishing. An alternative in situ cleaning method, using high-voltage oxygen conversion, was tested and shown to be mechanically effective but was not implemented in the automated protocol. The oxygen plasma cleaning introduces large mass-independent oxygen isotope memory effects into the reactor, presumably deposited as polymers forming on the glass walls of the reactor.^37^

### Laser Spectroscopy Triple Oxygen Isotope Analysis

The CO_2_ generated from the sample was introduced into a fully automated, custom-built dual-inlet system coupled to a laser spectrometer (Aerodyne Research Inc. TILDAS), enabling the analysis of the ^16^O^12^C^16^O, ^16^O^12^C^17^O, and ^16^O^12^C^18^O isotopologues.^30^ The inlet system diluted the CO_2_ analyte of both the sample and the working standard with CO_2_-free air to 420(±1) μmol mol^–1^. Analytical conditions, including cell temperature, electronics temperature, cell pressure, and analyte *p*CO_2_, were kept constant across multiple replicate analyses.^38^ Each replicate measurement consisted of 10 sample cycles bracketed by measurements of a working standard and was completed in about 2 h (including gas insertion, equilibration, and measurement). Measurement stability was evaluated by analyzing two in-house CO_2_ reference gases (heavy CO_2_ and light CO_2_) after every four samples.

The isotopic compositions of the working standard and the two reference gases were calibrated against VSMOW2 (*n* = 9) and SLAP2 (*n* = 7) waters using the TORCH method presented here. The resulting assigned values were δ^18^O = 28.048‰ and Δ′^17^O = −90 per meg for the working reference gas, δ^18^O = 80.424‰ and Δ′^17^O = −87 per meg for the heavy CO_2_, and −1.835‰ and −140 per meg for the light CO_2_. These values differ markedly from those previously assigned to these internal standards,^39^ which were anchored to the NBS-18 and IAEA-603 carbonate reference materials using fluorination-based reference values.^6^ Differences in the scaling of unknowns when using these newly assigned light and heavy CO_2_ values can be as large as 4‰ in δ^18^O and 140 per meg in Δ′^17^O (Figure S6).

### Data Evaluation

Measured δ^18^O and Δ′^17^O values for each sample, prepared and analyzed with the TORCH workflow, were corrected using a two-step process managed by a custom Python graphical interface, ensuring traceable and reproducible handling of raw and metadata files.

First, sample triple oxygen isotope values (δ^17^O, δ^18^O) were anchored to the VSMOW-SLAP scale using a two-point correction following Schoenemann et al. and Sharp and Wostbrock.^15,17^ The two-point correction is based on bracketing heavy and light CO_2_ working gas measurements, each preassigned using the TORCH calibration to VSMOW2 and SLAP2. Second, a correction was applied to account for oxygen isotope fractionation observed during glow discharge CO-to-CO_2_ conversion.^16^ Though conversion yields approach 100%, a small fraction of CO remains unconverted (due to insufficient pressure to sustain the glow discharge below 0.02 – 0.03 mbar). This correction is typically about 1.5‰ for δ^18^O and less than 10 per meg for Δ′^17^O at CO-to-CO_2_ conversion yields near 99%, as detailed further in the Results and Discussion.

## Results and Discussion

### Yield of the Oxygen Extraction

High-temperature TC/EA reduction oxygen extraction yields obtained by the TORCH method were generally at or above stoichiometric expectations across all solid standards. Absolute TC/EA yields for water are not provided due to uncertainties in water volume determination, which result from variability in capsule mass and geometry relative to the water mass. For the Barite standards, mean CO yields—calculated as the ratio of measured to expected CO input pressure in the discharge reactor—were 102 ± 1% for IAEA-SO-5, 107 ± 1% for IAEA-SO-6, and 102 ± 2% for UG-SO-1 when using the SiC outer tube (Figure 2). The benzoic acid and cellulose standards yielded a quantitative CO recovery of 100 ± 1%. In contrast, the first four IAEA-SO-5 samples processed with an Al_2_O_3_ ceramic tube exhibited higher apparent CO yields (∼109%), attributable to increased CO blank formation from high-temperature interaction of glassy carbon with oxygen from the ceramic material at 1450 °C (Figure S3). The transition to a SiC reactor effectively minimized this source of blank.

**Figure 2:**
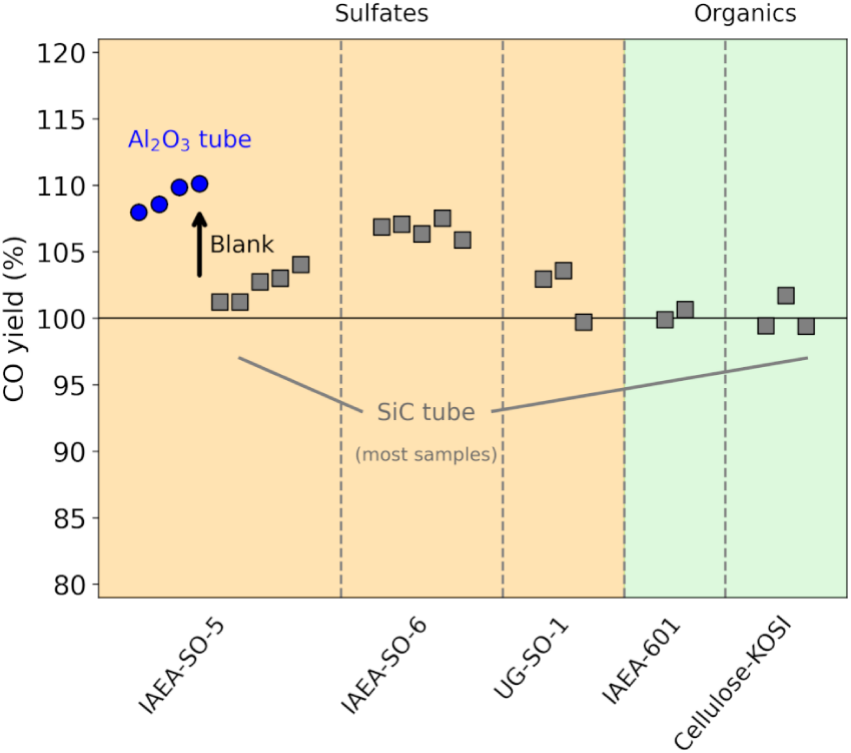
CO yield of solid standards obtained via high-temperature reduction using TC/EA. Blue circles indicate samples prepared with an Al_2_O_3_ ceramic outer tube, while gray squares represent samples reduced using a SiC outer tube in the TC/EA system.

The slightly elevated yields in Barite samples are attributed to residual moisture content in the standards despite storage in a desiccator and overnight heating at 50 °C. A thermogravimetric analysis (TGA) has been conducted for the three Barite standards (Figure S7). The TGA verified water contents of 2–5% with IAEA-SO-6 showing the largest moisture concentration. This is consistent with previous findings indicating that drying at 60 °C leaves 2–7% residual water in Barites.^40^ Complete removal of bound water may require higher pretreatment temperatures. Even after heating to 250 °C, 2% moisture was still observed in some Barite samples,^16^ while effective drying at 600 °C has been recommended elsewhere.^40^ Heated degassing under vacuum and handling of the samples under an inert noble gas atmosphere, as successfully applied for hydrogen isotope studies,^41,42^ could be adapted for the TORCH protocol in the future. The present results highlight the crucial influence of both reactor material selection and sample pretreatment on achieving quantitative yields.

### Triple Oxygen Isotope Composition of Standard Materials

The triple oxygen isotope compositions of the international and in-house reference standards determined using the TORCH method are summarized in Table 1 and illustrated in Figure 3. Observed values for δ^18^O ranged from −55.5‰ to 28.05‰, and for Δ′^17^O from −399 per meg to 20 per meg, encompassing the spectrum of commonly analyzed terrestrial materials.^43^

**Table 1:** Triple Oxygen Isotope Compositions of International Reference and In-House Standard Materials Acquired Using the TORCH Method^a^

**Sample**	**Material**	**δ** ^**17**^ **O (‰)**	**1 SD**	**SE**	**δ** ^**18**^ **O (‰)**	**1 SD**	**SE**	**Δ′** ^**17**^ **O (per meg)**	**1 SD**	**SE**	***n***
IAEA-SO-5	Barite	6.40	0.18	0.06	12.77	0.34	0.11	–324	13	4	9
IAEA-SO-6	Barite	–6.08	0.16	0.07	–10.97	0.33	0.15	–270	21	10	5
UG-SO-1	Barite	3.35	0.16	0.09	6.75	0.33	0.19	–204	16	9	3
IAEA-601	Benzoic Acid	11.41	0.19	0.14	22.49	0.40	0.28	–399	18	12	2
Cellulose-KOSI	Cellulose	14.56	0.22	0.13	28.05	0.44	0.26	–150	16	9	3
USGS46	Water	–15.71	0.19	0.11	–29.59	0.34	0.20	20	13	7	3
VSMOW2	Water	=0	0.15	0.05	=0	0.28	0.09	=0	10	3	9
SLAP2	Water	=–29.71	0.36	0.14	=–55.5	0.67	0.25	=–11	12	4	7

a The data is reported on the VSMOW-SLAP scale, the Δ′^17^O relative to a reference line with a slope of 0.528 and zero intercept. The “=” denotes assigned values.

**Figure 3:**
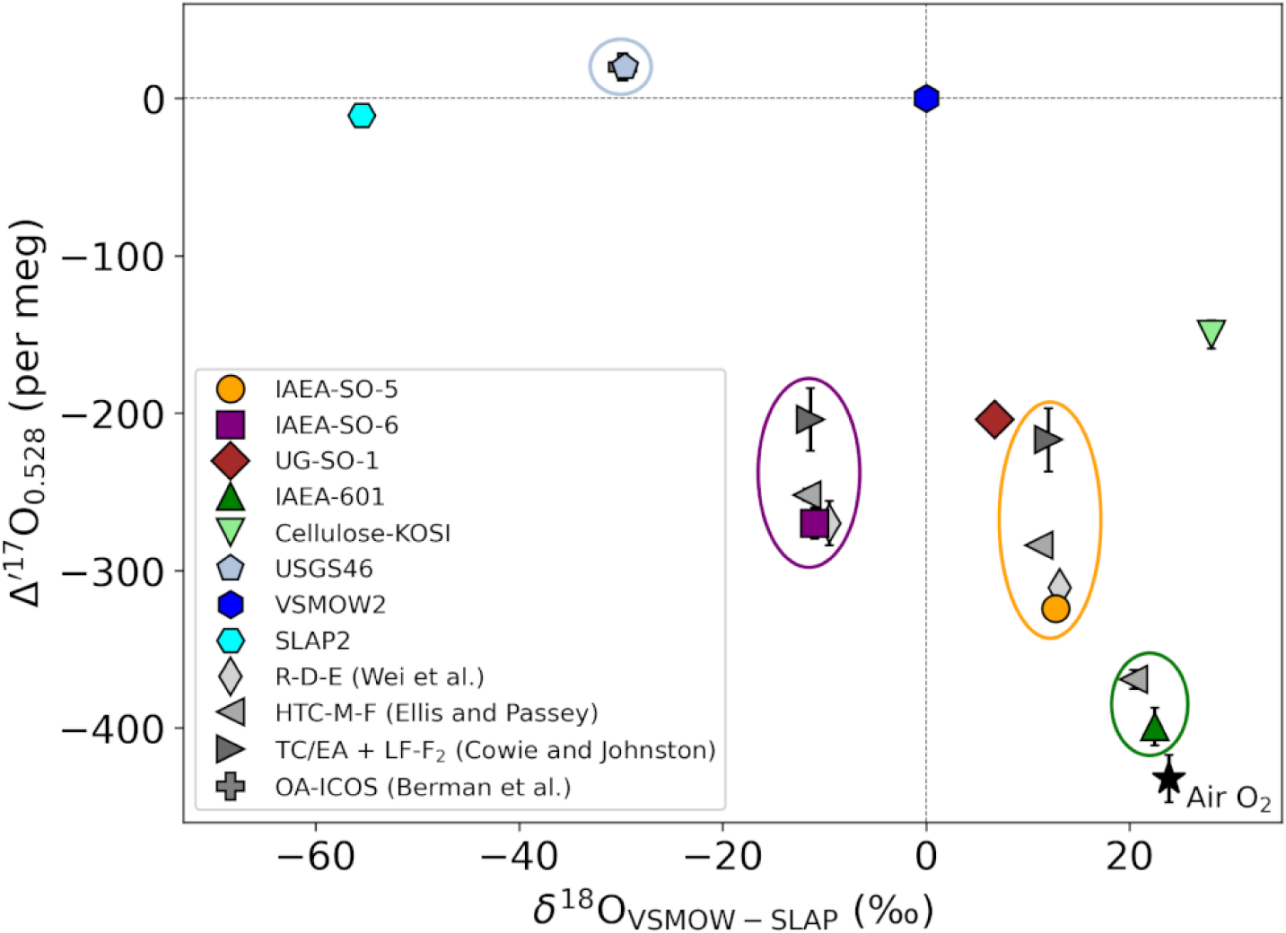
The triple oxygen isotope composition of international and in-house reference materials was determined using the TORCH method. Gray symbols represent previously reported values for the same standards of the color-coded ellipse they are included in. The TORCH USGS46 value (steel blue pentagon) overlaps the literature value (gray cross). The abbreviations R-D-E (Reduction, Discharge, and Exchange), HTC-M-F (High-Temperature Conversion, Methanation, and Fluorination), TC/EA + LF-F_2_ (TC/EA for δ^18^O and Laser Fluorination using F_2_ for Δ′^17^O), and OA-ICOS (Off-Axis Integrated Cavity Output Spectroscopy) refer to the respective methods.^9,14–16^ The air O_2_ value (black star) is displayed as a reference.^21^

The overall reproducibility for Δ′^17^O was typically ±11 per meg for water, ±14 per meg for Barite (excluding the higher value of ±21 per meg for IAEA-SO-6), and ± 17 per meg for organics. For IAEA-SO-6, the elevated uncertainty in Δ′^17^O is probably related to its elevated residual water content, indicated by the higher-than-stoichiometric oxygen yield for IAEA-SO-6 (Figure S8) with approximately 5% more water present (Figure S7) than in the other Barite standards.

Standard errors for five replicates were approximately ±7 per meg in Δ′^17^O, indicating that the precision of the TORCH method for sulfates, organics, and water is well suited for triple oxygen isotope studies in Earth and environmental sciences, which generally require a precision better than ±10 ppm.

In most cases, the δ^18^O reproducibility ranged between 0.3 and 0.4‰, except when compromised by memory effects. Notably, the lowest δ^18^O standard, SLAP2, displayed greater uncertainty (0.67‰) compared with the average reproducibility of 0.31‰ for the other water standards. This increased uncertainty can be attributed to a memory effect in the TC/EA system, which is more pronounced at low δ^18^O values (Figure S9). To mitigate this, the initial aliquots of water were routinely discarded, but some residual effect remained. Additionally, because SLAP2 differs by over 80‰ in δ^18^O from the working reference gas, this contrast likely contributed to reduced reproducibility in the TILDAS measurements for this standard.

The Δ′^17^O reproducibility of light CO_2_ and heavy CO_2_ internal standards in this study (±15–20 per meg; Figures S8 and S9) was slightly higher than that observed for the Göttingen TILDAS system previously (±10 per meg).^30^ The sample preparation process did not increase the uncertainty in Δ′^17^O, maintaining an average reproducibility of ±14 per meg. However, δ^18^O reproducibility for the CO_2_ reference gases was ± 0.023‰, significantly lower than the overall study uncertainty, suggesting that preparation steps mainly affected δ^18^O and had little impact on Δ′^17^O. Such mass-dependent effects are well known and previously described for triple oxygen isotope workflows,^27,44,45^ placing the reported reproducibility within the range documented for high-temperature conversion methods used in coupled δ^18^O and Δ′^17^O analyses.^14,27^

Sources of δ^18^O uncertainty include TC/EA reduction (memory effects), sample heterogeneity, the glow discharge step, and corrections for incomplete CO-to-CO_2_ conversion. The glow discharge conversion and related correction contributed an uncertainty of 0.22‰ in δ^18^O, based on repeated bottled CO gas tests (see Glow Discharge Fractionation Dynamics). Conventional high-temperature TC/EA reduction for δ^18^O analysis achieves ∼0.2‰ reproducibility for ∼200 μg samples.^46^ We speculate that using ten times larger sample masses here increases memory effects in the TC/EA, leading to the higher δ^18^O uncertainty observed (0.3–0.4‰), especially for water samples.

For all tested matrices, the TORCH method provided results that are consistent with established reference values (Table 2), provided that quantitative oxygen extraction was achieved. The USGS46 values matched closely with those reported by Berman et al.^9^ within 0.24 ± 0.34‰ for δ^18^O and 0 ± 13 per meg for Δ′^17^O and the USGS-certified δ^18^O^47^ within 0.21 ± 0.34‰, confirming the accuracy of the VSMOW-SLAP scaling for aqueous samples.

**Table 2:** Triple Oxygen Isotope Composition of International Reference Materials from Previous Studies Compared with the TORCH Method Presented Here^a^

**Sample**	**δ** ^**18**^ **O**_**VSMOW‑SLAP**_ **(‰)**	**Δ′** ^**17**^ **O**_**0.528**_ **(per meg)**	**Reference**	**Method**	**TORCH Δδ** ^**18**^ **O (‰)**	**TORCH ΔΔ′** ^**17**^ **O (per meg)**
*Barite*						
IAEA-SO-5	13.15 ± 0.10	–311 ± 7	Wei et al., 2024	R-D-E	–0.38 ± 0.35	–13 ± 15
IAEA-SO-5	11.11 ± 0.20	–284 ± 3	Ellis and Passey, 2023	HTC-M-F	1.66 ± 0.39	–40 ± 13
IAEA-SO-5	12.07 ± 0.02	–217 ± 20	Cowie and Johnston, 2016 (Δ′^17^O corrected by Sharp and Wostbrock, 2021)	TC/EA + LF-F_2_	0.70 ± 0.34	–107 ± 24
IAEA-SO-5	12.13 ± 0.33		Brand et al., 2009	TC/EA	0.64 ± 0.47	
IAEA-SO-6	–9.47 ± 0.22	–270 ± 14	Wei et al., 2024	R-D-E	–1.5 ± 0.4	0 ± 25
IAEA-SO-6	–11.71 ± 0.32	–252 ± 4	Ellis and Passey, 2023	HTC-M-F	0.74 ± 0.46	–18 ± 21
IAEA-SO-6	–11.36 ± 0.02	–204 ± 20	Cowie and Johnston, 2016 (Δ′^17^O corrected by Sharp and Wostbrock, 2021)	TC/EA + LF-F_2_	0.39 ± 0.33	–66 ± 29
IAEA-SO-6	–11.35 ± 0.31		Brand et al., 2009	TC/EA	0.38 ± 0.45	
*Benzoic acid*						
IAEA-601	20.42 ± 0.35	–369 ± 6	Ellis and Passey, 2023	HTC-M-F	2.07 ± 0.53	–30 ± 19
IAEA-601^c^	23.14 ± 0.19		Brand et al., 2009	TC/EA	–0.65 ± 0.44	
*Cellulose*						
Cellulose-KOSI (Sigma-Aldrich)	27.6 ± 0.3		Epstein et al., 1977	Ni-D and HgCl_2_-D	0.45 ± 0.54	
*Water*						
USGS46^c^	–29.80 ± 0.03		Coplen et al., 2013	Eq-MS	0.21 ± 0.34	
USGS46	–29.83 ± 0.03	20 ± 2	Berman et al., 2013	OA-ICOS	0.24 ± 0.34	0 ± 13
VSMOW2	0	0	IAEA defined		=0	=0
SLAP2^c^	–55.5	–11 ± 6	IAEA defined (δ^18^O) and Sharp and Wostbrock, 2021	Multiple	=0	=0

a The TORCH Δ values are defined as the offset of the data analyzed with the method used here from the reported literature values. The “=” denotes that TORCH values are scaled relative to the reference values. The superscript “c” denotes IAEA or USGS certified δ^18^O values. The method abbreviations are R-D-E (Reduction, Discharge, and Exchange), HTC-M-F (High Temperature Conversion, Methanation, and Fluorination), TC/EA + LF-F_2_ (TC/EA for δ^18^O and Laser Fluorination using F_2_ for Δ′^17^O), Ni-D and HgCl_2_-D (Ni and HgCl_2_ oxygen extraction with glow Discharge conversion and CO_2_ mass spectrometry), Eq-MS (H_2_O–CO_2_ Equilibration with dual inlet Mass Spectrometry), and OA-ICOS (Off-Axis Integrated Cavity Output Spectroscopy). Reference uncertainties are 1 SD and TORCH Δ uncertainties are propagated errors.

Barite standards analyzed with TORCH showed Δ′^17^O in excellent agreement with values reported by Wei et al.,^16^ with accordance within −13 ± 15 per meg (IAEA-SO-5) and 0 ± 25 per meg (IAEA-SO-6), and δ^18^O results aligned within uncertainty with the online TC/EA calibrations across multiple laboratories reported in Brand et al.^36^ Systematic positive offsets in Δ′^17^O (up to +107 per meg) and notable shifts in δ^18^O (up to 1.66‰) were observed in data from F_2_ laser fluorination^13,15^ and the HTC-M-F protocol.^14^ We speculate that systematic variations in δ^18^O and Δ′^17^O values between the different methods (Figure 3) mainly reflect the impact of incomplete oxygen yield during individual extraction or conversion steps to the final molecular configuration.

For organic reference materials (benzoic acid IAEA-601 and cellulose), the TORCH method yielded results that fall within the expected range for these standards. The Sigma-Aldrich commercial cellulose standard Cellulose-KOSI aligns with previously reported δ^18^O values^48^ within 0.45 ± 0.54‰. For benzoic acid IAEA-601, the measured δ^18^O value is 0.65‰ lower than that reported by Brand et al.,^36^ which is close to agreement when considering propagated errors. Minor amounts of undetected moisture in the benzoic acid may have shifted the measured δ^18^O to values lower than those certified. The HTC-M-F method^14^ yields a lower δ^18^O (by −2.07‰) and higher Δ′^17^O (by 30 per meg) for IAEA-601 compared to the TORCH results, reflecting the same trend of systematic differences observed for Barite standards. IAEA-601, produced via oxidation of toluene with atmospheric oxygen,^49^ should retain the Δ′^17^O anomaly of air O_2_ (−432 ± 15 per meg^21^) in the product. The Δ′^17^O value for IAEA-601 measured by TORCH is 33 per meg higher compared to the value reported for air O_2_ ^21^ but within error. The slightly lower δ^18^O of IAEA-601 compared to air O_2_ implies some limited fractionation in the oxidation process, the details of which are unknown to us. However, the coherence between our IAEA-601 measurement and air-induced O_2_ serves as a useful check on the integrity of VSMOW-SLAP scaling for organics.

In summary, by ensuring quantitative oxygen extraction, minimizing residual water, especially in hydrous Barite standards, and employing consistent VSMOW-SLAP scaling, the TORCH method delivers high-precision and high-accuracy triple oxygen isotope data across all analyzed matrices.

### Glow Discharge Fractionation Dynamics

Achieving a quantitative yield in all sample oxygen extraction and conversion processes is crucial to accurately determining triple oxygen isotope ratios. While TC/EA-based reduction is demonstrated to yield nearly quantitative extraction of sample oxygen as CO (Figure 2), the subsequent conversion of CO to CO_2_ using high-voltage glow discharge introduces measurable isotope fractionation, even with typical conversion yields of ∼99%^16^ (Figure 4). This effect arises from the minimum pressure threshold (∼0.02 mbar) required to sustain the glow discharge, preventing full conversion at very low CO pressures.

**Figure 4:**
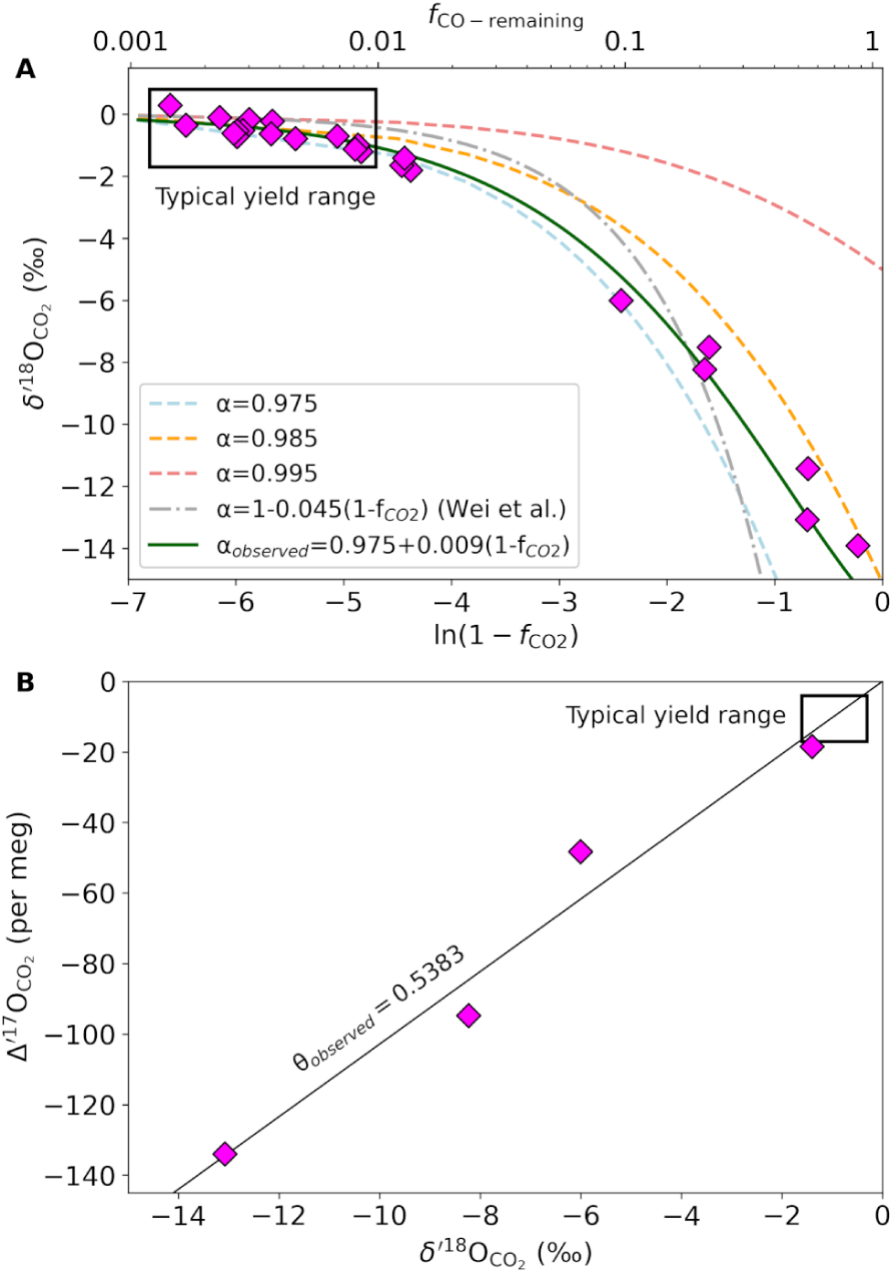
Oxygen isotope fractionation introduced during high-voltage glow discharge conversion of CO to CO_2_ for (A) δ^18^O and (B) Δ′^17^O. The Rayleigh-type fractionation trend can be explained with the fractionation factor ^18^α_observed_ = 0.975 + 0.009­(1 – fCO_2_) depicted with a green solid curve (A) and the fractionation exponent θ_observed_ = 0.5383 illustrated with a black solid line (B).

To evaluate this behavior, we systematically varied the CO-to-CO_2_ conversion yield using bottled CO gas and measured the resulting oxygen isotope fractionation (Figure 4). We observed a ∼14‰ difference in δ^18^O between 20% and near-quantitative CO_2_ yield, following a Rayleigh-type fractionation trend in excellent agreement with prior work by Wei et al.^16^ The observed fractionation factor ^18^α_observed_ = 0.975 + 0.009­(1 – fCO_2_) decreased slightly as the fraction of unconverted CO declined, and the trend for Δ′^17^O (θ_observed_ = 0.5383) was higher than the mass-dependent exponent reported by Wei et al.^16^ (θ = 0.5165), pointing to different oxygen isotope fractionation dynamics in the glow discharge reactor between the two setups. Variations in the instrument geometry, electrode configuration, and distance of the reaction zone to the LN_2_-cooled collection surface could all affect this exponent. Temperature gradients and corresponding thermal diffusion (Soret effect) have been shown to induce significant isotope fractionation,^50^ including unusual triple oxygen isotope slopes.^29^ The two different trends observed for Δ′^17^O are thus not unexpected.

For CO-to-CO_2_ conversion yields generally approaching 99%, the corrections applied to account for fractionation in Δ′^17^O are typically less than the reproducibility (<10 per meg). Nonetheless, our findings highlight the necessity for strict reproducibility in the setup and regular monitoring of fractionation effects, especially when any modifications are made to the experimental configuration. The automation of TORCH supports this reproducibility and robust procedural control.

We also conducted experiments with high-voltage O_2_-to-CO_2_ conversion with a carbon-coated reactor anode that further revealed Rayleigh fractionation behavior (Figure S10). Here, the δ^18^O fractionation was approximately 19‰ between 56% and near-quantitative CO_2_ yield ^18^α_observed_ = 0.968, about twice the magnitude observed for CO, in accordance with reaction stoichiometry. The triple oxygen isotope exponent θ_observed_ was 0.5122, with an outlier displaying strongly negative Δ′^17^O. The outlier is likely due to the transient formation and LN_2_-trapping of ozone,^51^ a species characterized by large positive Δ′^17^O anomalies^52^ that are counterbalanced by negative Δ′^17^O signatures of the produced CO_2_. Such findings underscore the complexity of discharge fractionation dynamics, especially if glow discharge O_2_ conversion is considered as an alternative to platinum-catalyzed methods.^53^

### Potential and Limitations of the TORCH Method

The TORCH method represents a significant step forward in triple oxygen isotope analysis by providing high accuracy, reproducibility, and efficient sample preparation for a range of materials including sulfates, water, and organics. Its strengths lie in its full automation and standardized preparation for both solids and liquids, which decrease operator error and increase sample throughput.

Accurate scaling to the VSMOW-SLAP scale of all analyzed materials is critical for interlaboratory comparisons, providing the basis for any reliable interpretation of the data. Our results emphasize that consistent triple oxygen isotope values can only be achieved when quantitative oxygen extraction of the material in question and direct scaling to VSMOW2 and SLAP2 measurements using the same analytical approach are assured.

While TORCH is already validated for sulfates, waters, and organics, adaptation for other minerals such as carbonates or phosphates simply requires adopting published techniques for respective TC/EA δ^18^O analyses from the literature. The method’s flexibility and modular design allow for simple implementation of any proven TC/EA protocol. Future expansion to include analyses of other gases from the same sample is also possible. For example, the hydrogen gas fraction released during TC/EA could enable δ^2^H analyses in future configurations (Figure S2). The H_2_ peak, separated chromatographically from CO, could be readily measured using a conventional isotope ratio mass spectrometer setup.^54^ Although not yet implemented in this study, it highlights the broader applicability of the TORCH method beyond triple oxygen isotopes.

Halas et al.^23^ originally developed the glow discharge technique, which was later adapted for Δ′^17^O measurements by Wei et al.,^16^ achieving a precision of 0.1‰ for δ^18^O in sulfate samples, a level of precision not yet attained with our current setup. Ongoing technical improvements such as optimizing reactor design, electrode cleaning routines, and sample handling with a focus on reducing memory effects are expected to yield further gains in analytical precision and robustness. The TORCH method offers a versatile and scalable platform that does not require hazardous materials such as BrF_5_ or CoF_3_, which are generally used to convert sample oxygen to O_2_ gas for mass spectrometric analyses. The TORCH method not only improves the precision of triple oxygen isotope analyses in challenging sample types such as sulfates but also provides a foundation for accurately normalizing the measured data on the VSMOW-SLAP scale. These advancements open up a range of future developments in stable isotope geochemistry and environmental science.

## Conclusions

We have developed the fully automated TORCH method for triple oxygen isotope analysis, integrating consistent sample preparation, quantitative TC/EA oxygen extraction, high voltage glow discharge conversion, and high-precision laser spectroscopy. The protocol enables direct normalization to the VSMOW-SLAP reference scale through the measurement of VSMOW2 and SLAP2, allowing consistent reporting for sulfate, water, and organic standards. Through comprehensive benchmarking with international reference materials, we demonstrated the TORCH method achieves reliable accuracy and external reproducibility, with precision for Δ′^17^O comparable to or exceeding those of established techniques. Our investigation quantified isotope fractionation introduced during glow discharge conversion, allowing for targeted corrections that preserve analytical integrity. We identified targeted methodological refinements that further improve analytical precision, and indicated the potential for adapting the TORCH system to additional stable isotope systems, such as δ^2^H. These advances make TORCH a versatile tool for triple oxygen isotope analysis in a wide range of materials, enabling further exploration of geochemical and environmental processes with improved confidence.

## Supplementary Material







## Notes

Additional figures including: Photograph of the silver tube station; LabView user interface of the reduction and conversion line; Evolution of the TC/EA carbon monoxide background signal; Photograph of the glow discharge reactor; High voltage of the glow discharge reactor with chamber pressure; Scaling differences of the TILDAS between VSMOW2-SLAP2 and carbonate based scaling; Thermogravimetric analyses of sulfate standards; Triple oxygen isotopes versus TC/EA yield of solid standards; Triple oxygen isotopes of water with time; Glow discharge fractionation dynamics of O_2_ to CO_2_ conversion (PDF)
All oxygen isotope data generated, including raw to corrected, referenced sample data (Summary sheet) and raw TILDAS data of samples and light CO_2_ and heavy CO_2_ with individual measurement conditions (TILDAS data sheet) (XLSX)
All log files of the reduction line sample runs (ZIP)
