# Supplementary material for: A Unified Method for Triple Oxygen Isotope Analysis of Sulfate, Water, and Organics
Source: Anal Chem. 2026 Mar 6;98(11):8111–20. doi: 10.1021/acs.analchem.5c06406 (PMC13019427; doi:10.1021/acs.analchem.5c06406)
Supplement: Supplementary file 1 [file ac5c06406_si_001.pdf]

Supporting Information

A Unified Method for Triple Oxygen Isotope Analysis of Sulfate, Water, and Organics

Fabian Zahnow[1,2,*], Dingsu Feng[1], Andreas Pack[2], David Bajnai[2] and Daniel Herwartz[1]

[1] Institute of Geosciences, Ruhr University Bochum, Universitätsstr. 150, 44801 Bochum, Germany

[2] Geoscience Center, University of Göttingen, Goldschmidtstraße 1, 37077 Göttingen, Germany

* Corresponding author: Fabian Zahnow

Email: fabian.zahnow@ruhr-uni-bochum.de

Table of Contents

Supplementary Figures **S2**

Figure S1. The semi-automated silver tube filling and crimp/cutting station S2

Figure S2. National Instruments LabView graphical user interface S3

Figure S3. Evolution of the TC/EA carbon monoxide background signal S4

Figure S4. The glow discharge reactor of the reduction line S5

Figure S5. High voltage between the platinum electrodes of the glow discharge reactor S6

Figure S6. The difference in (A) $\delta^{18}O$ and (B) $\Delta'^{17}O$ between VSMOW2-SLAP2 and acid digestion carbonate based (NBS18, IAEA-603) scaling of the TILDAS S7

Figure S7. Thermogravimetric analyses of sulfate standards S8

Figure S8. The (A) $\delta^{18}O$ and (B) $\Delta'^{17}O$ versus TC/EA CO yield of solid standards S9

Figure S9. The evolution of (A) the $\delta^{18}O$ and (B) the $\Delta'^{17}O$ of water aliquots S10

Figure S10. Oxygen isotope fractionation introduced during high voltage glow discharge S11

Supplementary Figures

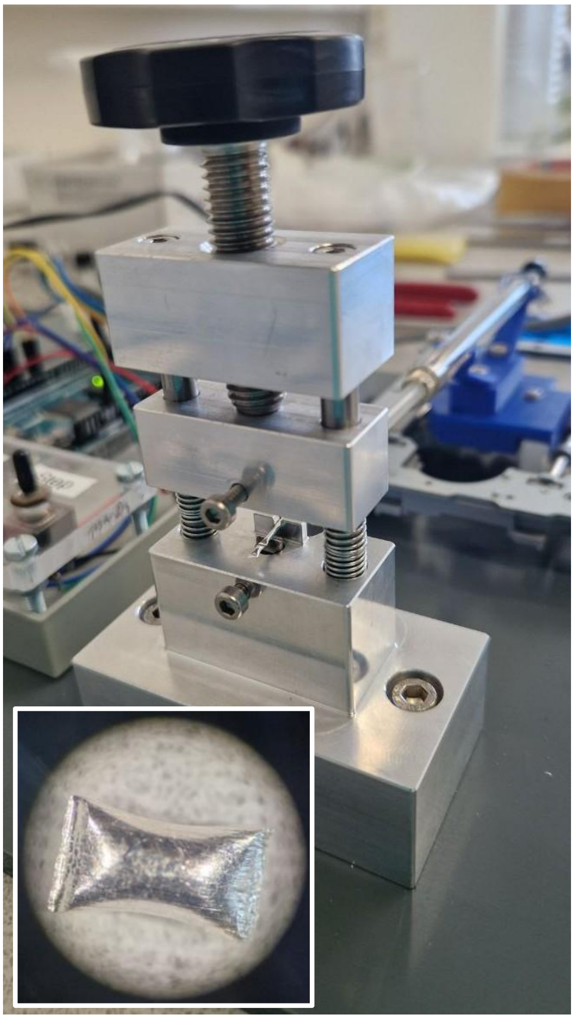

Figure S1. The semi-automated silver tube filling and crimp/cutting station for water samples at the University of Göttingen. A light microscope image of a 3 mm long filled and closed silver tube capsule is visible in the lower left corner.

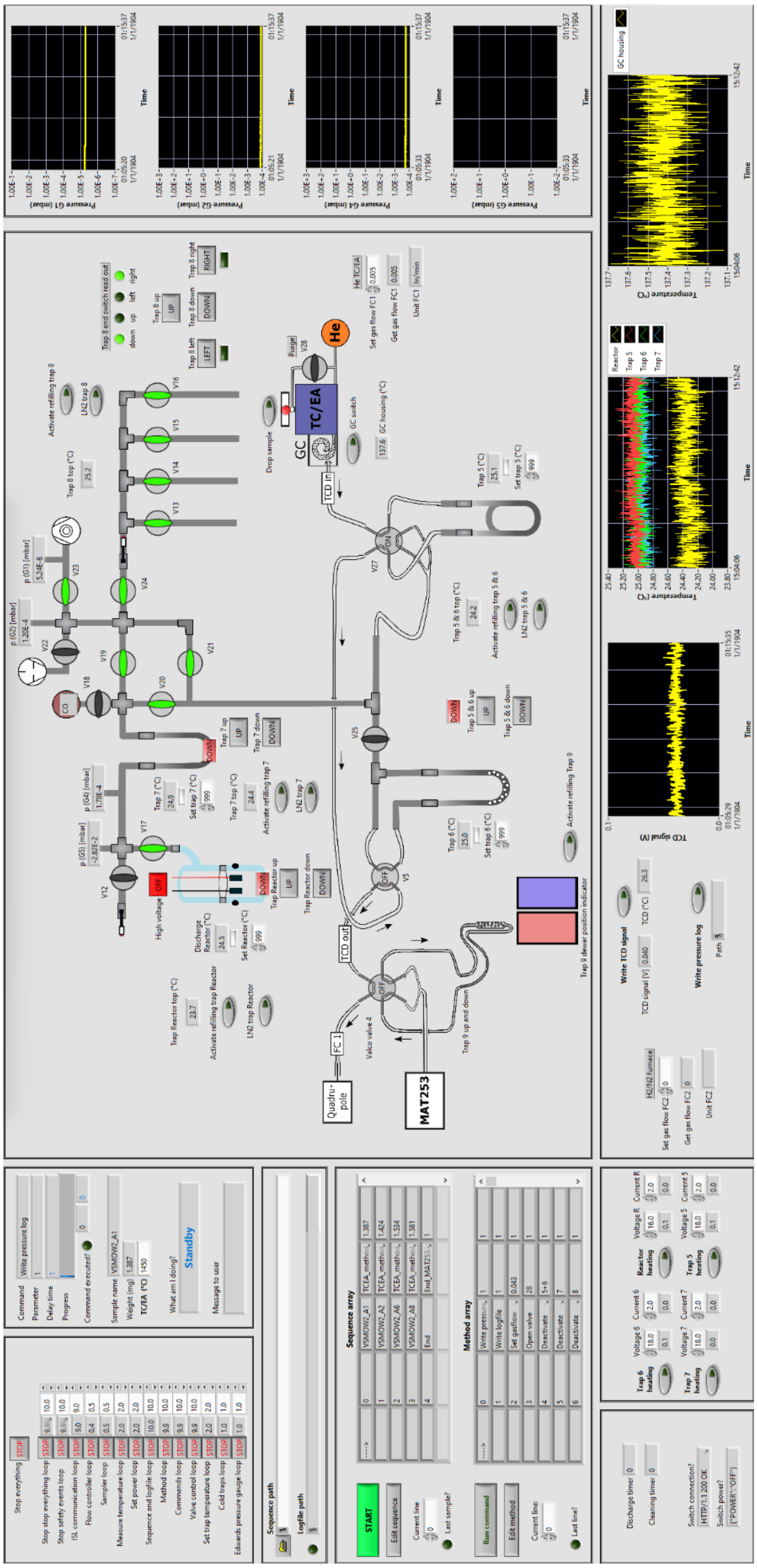

Figure S2. National Instruments LabView graphical user interface of the reduction and conversion unit of the TORCH at the University of Göttingen, Germany. Trap 9 and Valco Valve 4 are additional modifications for trapping the hydrogen peak released from samples in the TC/EA and were not used in this study.

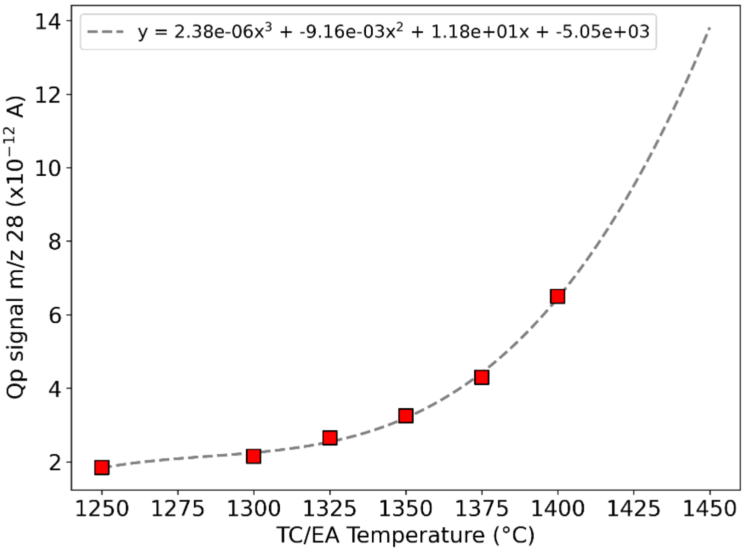

Figure S3. Evolution of the TC/EA carbon monoxide background signal – determined with the Quadrupole (Qp) mass spectrometer – with temperature, using a conventional Al_2O_3 outer tube.

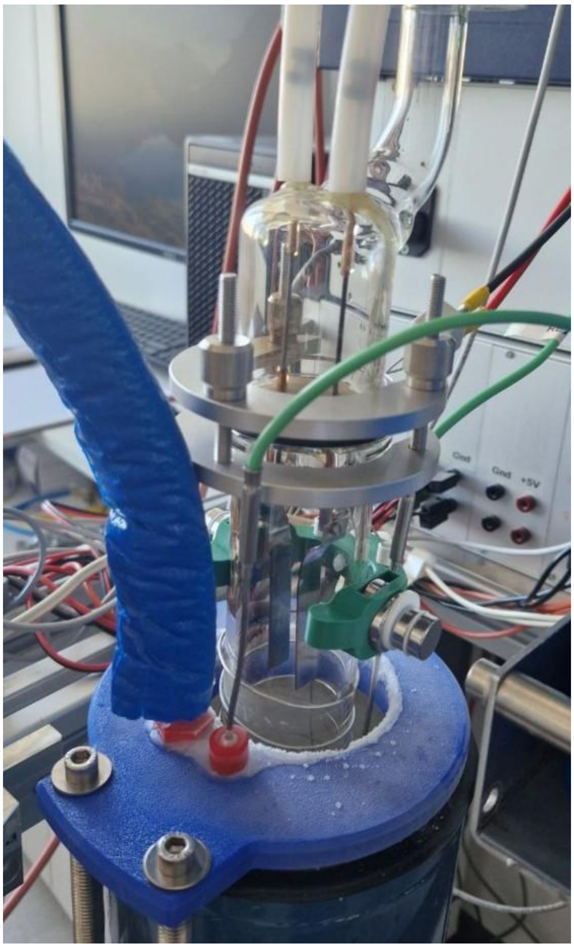

Figure S4. The glow discharge reactor of the reduction line at the University of Göttingen. The bottom part is immersed in LN_2 that is automatically refilled. Two neodym magnets at the outer wall of the reactor focus the electric discharge between the two platinum electrodes.

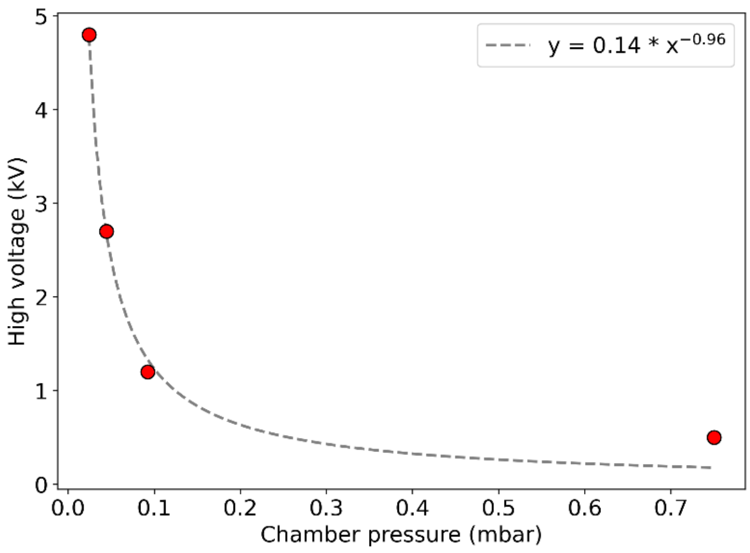

Figure S5. High voltage between the platinum electrodes of the glow discharge reactor at different chamber pressures.

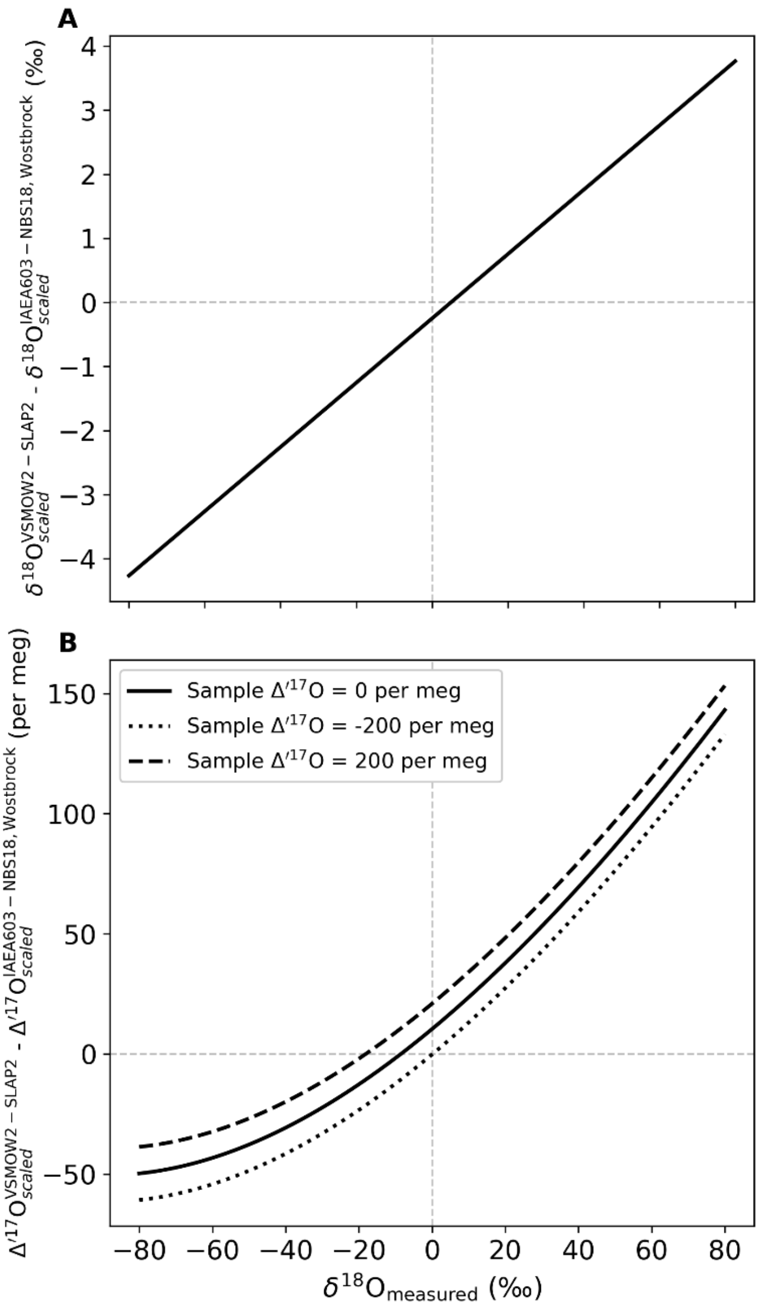

Figure S6. The difference in (A) $\delta^{18}O$ and (B) $\Delta'^{17}O$ between VSMOW2-SLAP2 and acid digestion carbonate based (NBS18, IAEA-603) scaling of the TILDAS reference gases at the University of Göttingen. The difference in $\Delta'^{17}O$ depends on the measured $\delta^{18}O$ and the $\Delta'^{17}O$ of the sample. A sample with a positive $\Delta'^{17}O$ (dashed line) will have a larger difference at higher $\delta^{18}O$ than a sample with a negative $\Delta'^{17}O$ (dotted line).

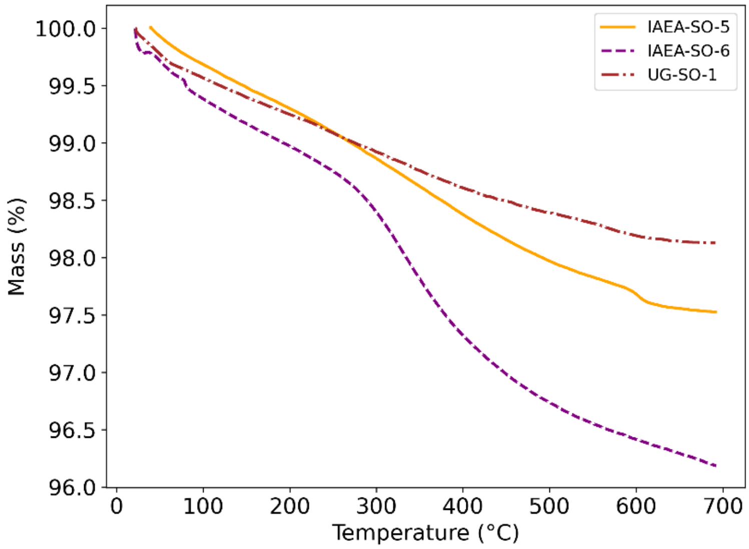

Figure S7. Thermogravimetric analyses of sulfate standards showing the release of moisture with elevated temperature.

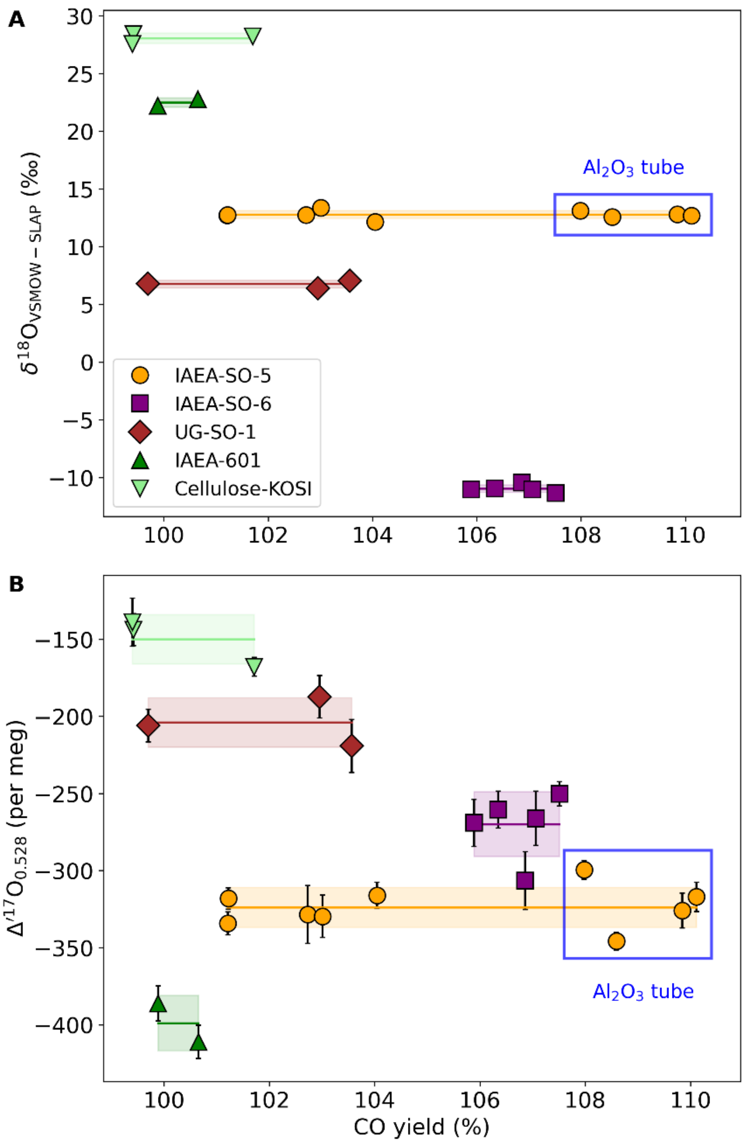

Figure S8. The (A) $\delta^{18}O$ and (B) $\Delta'^{17}O$ versus TC/EA CO yield of solid standards, determined with the TORCH approach. The legend in (A) is true for both plots. The blue rectangles indicate IAEA-SO-5 samples prepared with an Al_2O_3 ceramic TC/EA outer tube. The error bars represent the SE internal precision of the individual TILDAS measurements. For $\delta^{18}O$, the internal error (~0.015 ‰) is smaller than the markers. The average values (solid colored lines) and 1SD error envelopes are indicated for each sample.

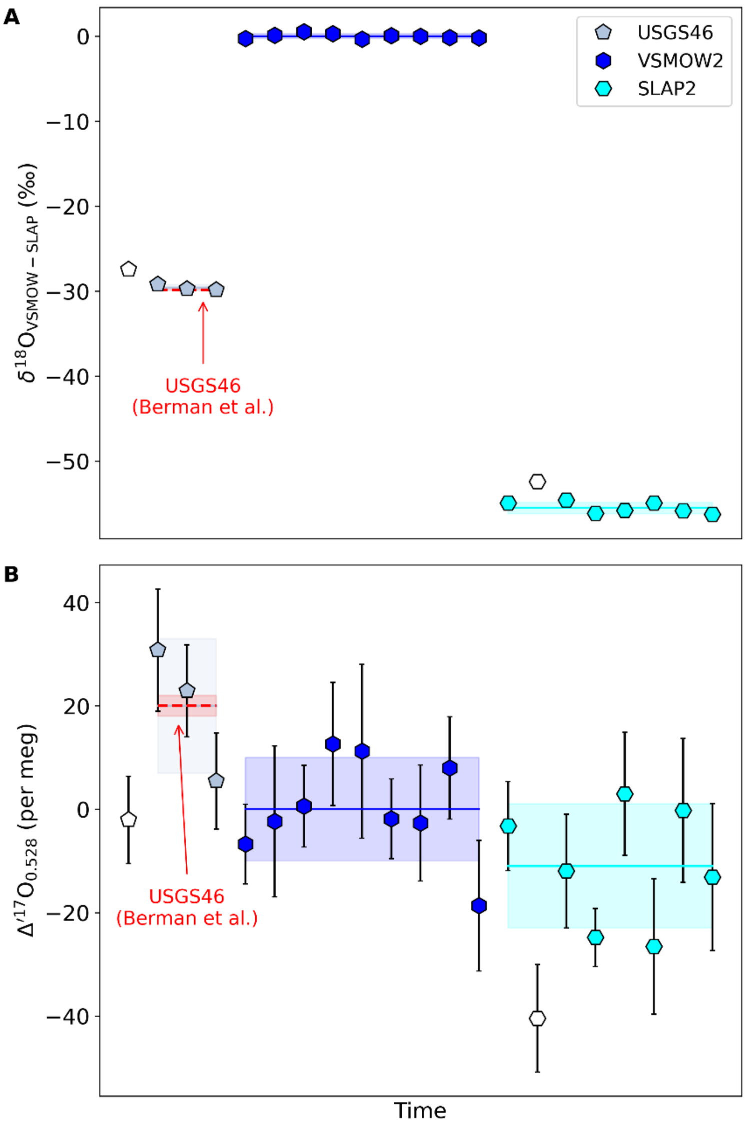

Figure S9. The evolution of (A) the $\delta^{18}O$ and (B) the $\Delta'^{17}O$ of water aliquots prepared with the TORCH method with relative time. The legend in (A) is true for both plots. Unfilled symbols represent discarded first aliquots afflicted with a strong memory effect. The error bars represent the SE internal precision of the individual TILDAS measurements. For $\delta^{18}O$, the internal error (~0.015 ‰) is smaller than the markers. The average values (solid colored lines) and 1SD error envelopes are indicated for each sample. The USGS46 triple oxygen isotope values from Berman et al. (Berman, E. S. F.; Levin, N. E.; Landais, A.; Li, S.; Owano, T. Measurement of δ18O, δ17O, and 17O-excess in water by off-axis integrated cavity output spectroscopy and isotope ratio mass spectrometry. *Analytical chemistry* **2013**, *85*, 10392–10398.) are shown as reference with red dashed lines and the respective 1SD error envelopes.

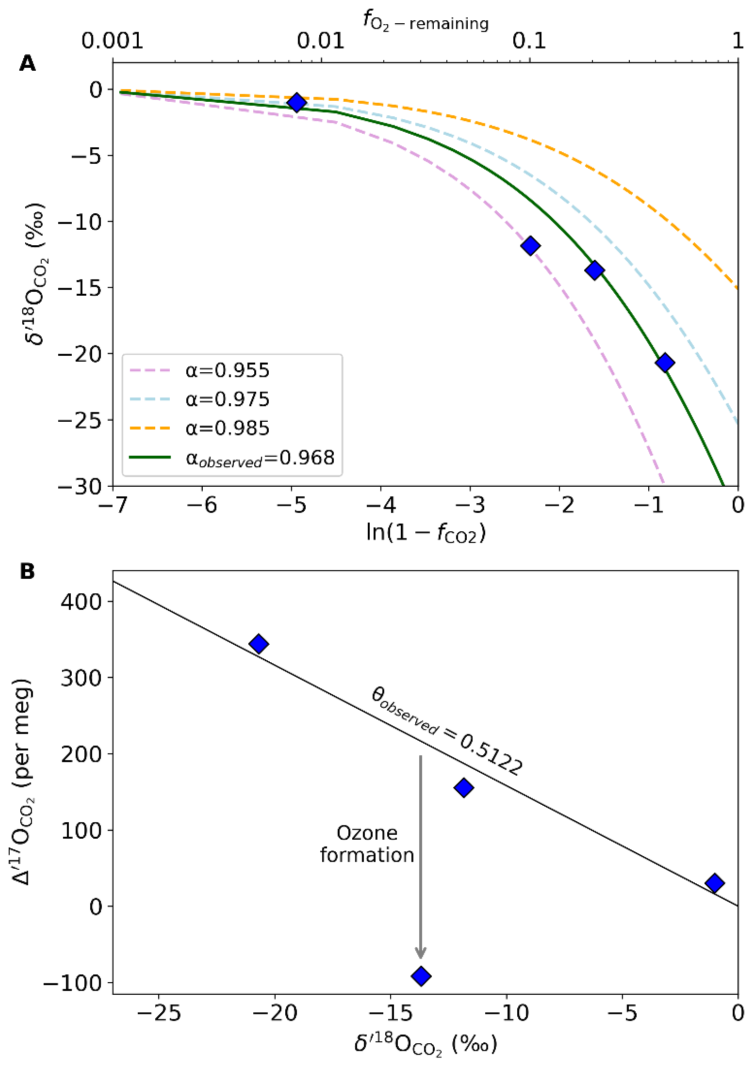

Figure S10. Oxygen isotope fractionation introduced during high voltage glow discharge conversion of O_2 to CO_2 for (A) $\delta^{18}O$ and (B) $\Delta'^{17}O$. The Rayleigh type fractionation trend can be explained with the fractionation factor $^{18}\alpha_{observed}$ = 0.968 depicted with a green solid curve (A) and the fractionation exponent $\theta_{observed}$ = 0.5122 illustrated with a black solid line (B). The outlier towards negative $\Delta'^{17}O$ is likely due to ozone formation during discharge.
